# Lucifer Match Making and Amorphous Phosphorus

**Published:** 1852-10

**Authors:** 


### CHEMISTRY.

*Lucifer Match Making and Amorphous Phosphorus.*—The announcement of Professor Shrotter's discovery of the mode of preparing amorphous phosphorus, derived much of its practical interest from the supposition that the phosphorus in this state would be less dangerous and injurious to the persons engaged in the manufacture of lucifer matches. A medal was awarded to Mr. Albright for the introduction of the prepared phosphorus as an article of commerce, and it may now be obtained at a moderate price of Messrs. Sturge, of Birmingham. The dreadful disease to which the makers of lucifer matches are liable, from inhaling the fumes of ordinary phosphorus, having been described and brought under public notice, it might have been supposed that no time would have been lost in ascertaining the value of the above discovery as a means of alleviating so much human suffering. Matches prepared with the amorphous phosphorus were shown in the Great Exhibition, and it was stated at the time that in the manufacture of these matches, the evils arising from the inhalation of deleterious fumes were obviated, while the result of the experiment was satisfactory.

It is, however, difficult to introduce any innovation of this kind into an extensive branch of manufacture. A series of experiments must be made to test the efficacy of the new preparations, the most advantageous mode of employing it, the quality of the goods, and the economy of the process. In the mean time the several departments of the manufactory are progressing like clock-work. All hands are busily employed, the proprietor is fully occupied with superintending the operations and the accounts, and a large box of amorphous phosphorus remains in the office unpacked, waiting for a convenient opportunity to complete the experiments.

Such was the state of affairs at Mr. Dixon's manufactory at Newton Heath, near Manchester, on the occasion of a recent inspection. Outside the building large piles of timber were stored up ready for use. A machine worked by a steam-engine was reducing blocks into the form of matches. A block previously cut the length of the match, and pressed against the side of the machine, disappeared in a few seconds. The sticks being removed into the next room were tied into bundles about eight inches in diameter, ready for dipping in sulphur. This was done in another room in an iron vessel over a furnace. Immediately after the dipping, the workman gives each bundle a slight pressure with a rotatory movement, to separate the matches from each other at the moment of solidification, otherwise the sulphur would cohere into a solid mass. The matches are next transferred into a room where they are arranged, so as not to be in contact with each other, in frames about two feet by one foot, ready for the phosphorus dipping. The composition used for this purpose consists of chlorate of potash, phosphorus, and glue, and it is spread in a thin layer on a stone or marble slab, heated below by steam or hot water. The operator holds the frame lengthways, and dips the ends of the matches in the composition, taking care that all of them are coated. Sometimes the sticks are in the first instance cut twice the required length, dipped at both ends, and afterwards bisected. In the process of cutting they occasionally ignite, occasioning loss and also vitiating the atmosphere. When the dipping is completed, they are taken to the sorting room and packed in boxes. In another room the boxes are labelled and sent to the packing room. The boxes are made on the premises, the shavings cut, and the tops and bottoms stamped by machinery, cut to the proper size, glued, and fitted, which operations are performed in separate apartments. Each box of lucifer matches, price retail one halfpenny, passes through the hands of seventeen persons, chiefly children. The Factory Act is not applicable to these establishments, and the children, averaging from seven to twelve years of age, work twelve and sometimes thirteen hours in the day. They earn (by piece work) from 3*s.* to 5*s.* a week, and the adults from 9*s.* to 12*s.*

The cases of disease occur chiefly in the phosphorus dipping room, sometimes in the room where the matches are sorted and packed in boxes, but seldom in other parts of the establishment. The nature of the disease is described in the *Dublin Quarterly Journal of Medical Science*, for August, page 1·0, by Mr. Harrison :

"An affection ensues which is so insidious in its nature that it is at first supposed to be common toothache, and a most serious disease of the jaw is produced before the patient is fairly aware of his condition. The disease gradually creeps on until the sufferer becomes a miserable and loathsome object, spending the best period of his life in the wards of a public hospital. * * * * Many patients have died of the disease; many, unable to open their jaws, have lingered with carious and necrosed bones; others have suffered dreadful mutilations from surgical operations, considering themselves happy to escape with the loss of the greater portion of the lower jaw."

Mr. Harrison's paper contains much interesting information, with the medical reports of several cases.

It would be foreign to our purpose to enlarge upon this view of the subject; but the disease being of chemical origin, the *modus aperandi* of the poison may involve a chemical enquiry. Does the phosphorus when inhaled destroy the vitality of the bone by chemical action on its substance? or does it operate merely as an irritant on the tissues, causing inflammatory action? The bone in its diseased state has a spongy cellular appearance, with excrescences of a similar character adhering to it. The teeth generally continue sound and white, while the jaw which contains them is altered in texture, dead, and discolored. We believe the diseased bone has not been chemically examined. Whether such examination would throw any light upon the subject is a speculative question; but we think it not unworthy of consideration.

There are at this time in the manufactory several persons who have suffered severely from the disease, and who on recovery immediately returned to their work—not however to the dipping department. In the Museum of the Manchester Infirmary is the lower jaw of a young woman who is now at work. Her face is much disfigured by the loss of her chin, and in looking into her mouth the root of the tongue is seen connected with her under lip, the space formerly occupied by the jaw being obliterated by the contraction of the check. A young man who has lost his jaw is also in the factory. They are not isolated cases.

It is stated in the factory that the workpeople have sometimes applied the phosphorus paste to decayed teeth, under the idea that it was a cure for the toothache, and to this imprudence some of the early cases of the disease are attributed. The frightful nature of the disorder is now sufficiently understood to serve as an incentive to greater precautions. Increased attention has been paid to ventilation and cleanliness, and the practice of taking meals on the premises is not allowed. It appears, however, from the statements of some of the workpeople who are engaged in the phosphorus dipping room, that their clothes become incandescent in the dark, and although the cases of the disease are less frequent than they have been formerly, a security against its recurrence is not attained. The proprietor of one factory states that he has had no cases in his establishment on account of a more careful method of dipping the matches, by which the face of the operator is further removed from the source of danger; but we are informed that some patients, from that factory have applied for medical relief in the neighborbood. Mr. Standring informs us that there is now in the Manchester workhouse a young woman suffering from a "phosphoric jaw." She worked three years in a match manufactory; she then went to a silk mill, where she had been about a year and a half before the disease first made its appearance. Eleven months since she was admitted into the infirmary and remained there eighteen weeks, since which time she has been an inmate of the workhouse. The disease at present affects only one side of the jaw—a portion of which is likely soon to be detached.

Various means of prevention have been tried, and others suggested. In a manufactory in Dublin, camphor is added to the composition, which masks the smell, and is said to act as a prophylactic. This latter opinion requires further proof. Mr. Taylor, of Nottingham, suggests the use of a mask with a tube communicating with the outside of the building. Mr. Stanley, of St. Bartholomew's Hospital, recommends the exposure of oil of turpentine in saucers about the workrooms, as a solvent of the fumes of phosphorus. Dr. Baur recommends the use of a sponge or handkerchief moistened with a solution of soda or potash and applied to the mouth. The proprietor of the factory above referred to states that he has diminished the quantity of phosphorus to less than a third of that which he formerly used, and that by this and other precautions the prevalence of the disease has been greatly diminished. He has tried the amorphous phosphorus on a small scale, by way of experiment, and says that it is more expensive than the ordinary kind, as a larger quantity is required. But the chief objection appears to be that the composition now in use answers quite well. The matches never fail; the mode of preparing the composition is understood; the result is known, and the demand for the matches unceasing. The amorphous phosphorus requires further trial; the makers are not yet accustomed to it. If it should fail their trade would be injured; the experiment would interfere with the habits of the factory; therefore the operations are continued in the usual way, the box of Sturge's phosphorus remains unopened in the office, and the value of the discovery is not fairly put to the test.—*London Pharmaceutical Journal, September*, 1852.

